# Singapore Tele-technology Aided Rehabilitation in Stroke (STARS) trial: protocol of a randomized clinical trial on tele-rehabilitation for stroke patients

**DOI:** 10.1186/s12883-015-0420-3

**Published:** 2015-09-05

**Authors:** Gerald Choon-Huat Koh, Shih Cheng Yen, Arthur Tay, Angela Cheong, Yee Sien Ng, Deidre Anne De Silva, Carolina Png, Kevin Caves, Karen Koh, Yogaprakash Kumar, Shi Wen Phan, Bee Choo Tai, Cynthia Chen, Effie Chew, Zhaojin Chao, Chun En Chua, Yen Sin Koh, Helen Hoenig

**Affiliations:** Saw Swee Hock School of Public Health, National University of Singapore, National University Health System, #10-03-G, Tahir Foundation Building, Block MD1, 12 Science Drive 2, Singapore, 117549 Singapore; Yong Loo Lin School of Medicine, National University of Singapore, National University Health System, Singapore, Singapore; Department of Electrical and Computer Engineering, National University of Singapore, Singapore, Singapore; Singapore General Hospital, Singapore, Singapore; National Neuroscience Institute, Singapore General Hospital campus, Singapore, Singapore; Ang Mo Kio Thye Hua Kwan Hospital, Singapore, Singapore; Department of Surgery, Medicine and Biomedical Engineering, Duke University, Durham, USA; Department of Rehabilitation Medicine, National University Hospital, Singapore, Singapore; Department of Biological Science, National University of Singapore, Singapore, Singapore; Investigational Medicine Unit, National University Health System, Singapore, Singapore; Physical Medicine & Rehabilitation Service, Durham Veterans Affairs Medical Centre, Durham, USA

**Keywords:** Tele-rehabilitation, Home rehabilitation, Stroke, Post-stroke functional recovery

## Abstract

**Background:**

Most acute stroke patients with disabilities do not receive recommended rehabilitation following discharge to the community. Functional and social barriers are common reasons for non-adherence to post-discharge rehabilitation. Home rehabilitation is an alternative to centre-based rehabilitation but is costlier. Tele-rehabilitation is a possible solution, allowing for remote supervision of rehabilitation and eliminating access barriers. The objective of the Singapore Tele-technology Aided Rehabilitation in Stroke (STARS) trial is to determine if a novel tele-rehabilitation intervention for the first three months after stroke admission improves functional recovery compared to usual care.

**Methods/design:**

This is a single blind (evaluator blinded), parallel, two-arm randomised controlled trial study design involving 100 recent stroke patients. The inclusion criteria are age ≥40 years, having caregiver support and recent stroke defined as stroke diagnosis within 4 weeks. Consenting participants will be randomized with varying block size of 4 or 6 assuming a 1:1 treatment allocation with the participating centre as the stratification factor. The baseline assessment will be done within 4 weeks of stroke onset, followed by follow-up assessments at 3 and 6 months. The tele-rehabilitation intervention lasts for 3 months and includes exercise 5-days-a-week using an iPad-based system that allows recording of daily exercise with video and sensor data and weekly video-conferencing with tele-therapists after data review. Those allocated to the control group will receive usual care. The primary outcome measure is improvement in life task’s social activity participation at three months as measured by the disability component of the Jette Late Life Functional and Disability Instrument (LLFDI). Secondary outcome variables consist of gait speed (Timed 5-Meter Walk Test) and endurance (Two-Minute Walk test), performance of basic activities of daily living (Shah-modified Barthel Index), balance confidence (Activities-Specific Balance Confidence Scale), patient self-reported health-related quality-of-life [Euro-QOL (EQ-5D)], health service utilization (Singapore Stroke Study Health Service Utilization Form) and caregiver reported stress (Zarit Caregiver Burden Inventory).

**Discussion:**

The goal of this trial is to provide evidence on the potential benefit and cost-effectiveness of this novel tele-rehabilitation programme which will guide health care decision-making and potentially improve performance of post-stroke community-based rehabilitation.

**Trial Registration:**

This trial protocol was registered under ClinicalTrials.gov on 18 July 2013 as study title “The Singapore Tele-technology Aided Rehabilitation in Stroke (STARS) Study” (ID: The STARS Study, ClinicalTrials.gov Identifier: NCT01905917).

## Background

Supervised therapy is an important factor in determining post-stroke functional recovery. In a 1-year cohort study on local post-stroke patients in the community, only a third of the subjects undergo supervised rehabilitation one month after discharge into the community [1]. Those who performed supervised therapy >25 % of the recommended amount of time recovered faster than those who performed a lesser amount of supervised therapy <25 % at 1- and 6-months. Performing therapy at outpatient rehabilitation centre at 1- and 6-months post-stroke is also independently predictive of better Barthel Index (BI) scores at 1 year (72.4 vs. 62.7 at 1 month and 74.7 vs. 59.4 at 6 months). Greater functional independence is associated with reduced caregiver burden, better patient health-related quality of life and potentially lower cost of care [2–5].

In a mixed methods study on non-adherence with recommendations for continued rehabilitation, although 86 % acknowledged that rehabilitation was beneficial, only 40 % intended to continue with rehabilitation after discharge, citing functional and social (e.g. unwillingness to inconvenience their caregiver) barriers [5]. Based on the qualitative results, a quantitative questionnaire was administered on a new set of 70 patients with follow-up telephone interviews at three, six, nine and twelve months after discharge. The longitudinal adherence to rehabilitation rate fell from 100 % in hospital to 20.3 % at 3 months, 9.8 % at 6 months, 6.3 % at 9 months and 4.3 % at 12 months after discharge.

Solutions to addressing non-adherence to rehabilitation include the use of transportation and mobility systems such as stair-lifts for stroke survivors living in apartments on floors without access to an elevator. Paid handicap-fitted transport services to ferry stroke survivors to and back from outpatient rehabilitation centers can also overcome physical barriers. Providing paid caregivers for social support and subsidies for outpatient rehabilitation may reduce financial and social barriers. Nevertheless, all such services and/or equipment are expensive; moreover, caregivers are in short supply around the world [5].

Given the heavy cost and inconvenience incurred in transporting stroke survivors to outpatient rehabilitation centers, home rehabilitation seems to be a viable option in the delivery of post-stroke recovery. Even through home rehabilitation was shown to be equally effective as inpatient rehabilitation [6, 7], it is less cost-efficient than outpatient rehabilitation center [5]. Gladman et al. randomized 327 patients to receive domiciliary or routine (hospital-based) care. Over a 14-month time frame, mean per patient costs for hospital-based services were less than domiciliary services [8]. Indeed, the cost of home rehabilitation is at least two times more expensive than therapy at an outpatient rehabilitation center (in Singapore, the norm cost of one hour of home rehabilitation is S$125 per hour and one hour of centre-based rehabilitation is S$50 per hour [5]). The relatively higher cost for home rehabilitation can be attributed to the greater economies of scale in outpatient rehabilitation as compared to home rehabilitation [8]. For instance, home therapists can only see one patient at a time whereas centre-based therapists can directly supervise the treatment of more than one patient at a time.

Recent studies focus on the use of home-based tele-health technologies to provide support and guidance by rehabilitation therapists at a distance, representing a novel potential approach for addressing the access to post-acute rehabilitation care problem for stroke survivors [9]. Tele-rehabilitation provides greater convenience to patients and families and encourages rehabilitation within the patient’s home. A study by Sanford et al. showed the feasibility of providing a multi-factorial, multidisciplinary individualized, home-based tele-rehabilitation intervention, demonstrating improved self-efficacy in mobility-impaired adults (n = 16) to the same degree as those who received supervised rehabilitation with physiotherapists and occupational therapists at outpatient rehabilitation centers (n = 16) [10]. These findings were corroborated and extended in two subsequent randomized clinical trials, with differing populations (stroke survivors and intensive care survivors) [11–13]. However, the intervention still require home visits and video-recordings by a therapy aide. Moreover, as video-recordings do not allow the collection of physical and sensor information from patients, this approach to tele-rehabilitation limits the therapists’ assessment of patient performance.

Given the current heterogeneity and room for improvements in solutions related to post-stroke rehabilitation, there is a need for well-designed intervention and rigorous trial to close the gap. The Singapore Tele-technology Aided Rehabilitation in Stroke (STARS) Study is a parallel, two-arm (intervention-control), single blind (evaluator-blind) randomised controlled trial to determine if a novel tele-rehabilitation intervention can improve functional recovery of post-stroke patients. For the tele-rehabilitation intervention arm of this study, the team developed small affordable lightweight wireless wearable sensors which can be strapped onto patients to allow real-time biofeedback and collection of sensor data from the sensors for subsequent transmission to patient’s rehabilitation team wirelessly via the internet. The sensor data is automatically summarized in chart form for quick review by therapist to allow them to prescribe further rehabilitation exercises remotely. The system also automatically allows the patient to self-record videos of them exercising which will also be transmitted to the therapist (termed ‘tele-therapist’) for review where needed, and allow patients and their therapists to communicate with each other using video-conferencing. The study’s primary hypothesis is that this novel tele-rehabilitation intervention during the first three months after stroke admission will result in greater functional recovery compared to usual care three months after stroke admission, as determined by the Jette Late Life Functional and Disability Instrument (LLFDI). Secondary hypotheses are that tele-rehabilitation intervention during the first three months after stroke admission will result in greater functional recovery as determined by gait speed, 2-min walk test and Shah modified Barthel Index, less health service utilization, greater contact time with therapist, better balance, better quality of life, less caregiver burden and be more cost-effective compared to usual care three months after stroke admission. Process measures in the form of a diary will be used to assess improved adherence in rehabilitation. Outcomes will also be assessed again at six months post-stroke to determine whether any improvement in outcomes is sustained in both groups.

## Methods/Design

### Ethical approval

All procedures conducted during this trial with human participants were carried out in compliance with institutional ethical standards. All research procedures were approved by an Institutional Review Board (IRB) at each participating site: Singapore General Hospital (SGH) from Centralised Institutional Review Board (CIRB Reference No. 2011/779/D), and Ang Mo Kio Thye Hua Kwan Hospital (AMKTHKH) from National University of Singapore (NUS) IRB (NUS IRB Reference Code 11–013) and accepted by the National Healthcare Group Domain Specific Review Board (DSRB). SGH is an acute hospital with an acute stroke unit and an inpatient rehabilitation unit, whereas AMKTHKH is an inpatient rehabilitation hospital.

### Study design

**Fig. 1:**
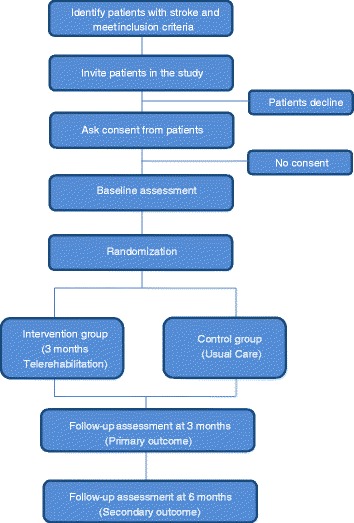
STARS Trial Workflow Diagram

This is a parallel, two-arm (intervention-control), and single blind (evaluator-blind) randomised controlled trial of a novel home-based tele-rehabilitation intervention provided for 3 months after discharge for stroke admission compared with usual care. Prior to the study, a pilot study was done to test the procedures, interventions and assessments. Eligible participants are randomized to the intervention and control groups at baseline before discharge from the hospital. With regards to mean length of stay in hospital, Chen. et al. reported that patients admitted in local inpatient rehabilitation hospitals (e.g. AMKTHKH) for stroke stayed for 33 days [14]. In another study done by Ng. et al., in which 57.9 % of the participants were admitted to an acute hospital (e.g. SGH) for stroke, the mean length of stay for acute referring unit and inpatient rehabilitation unit were 14.5 and 21.5 days respectively [15]. The primary outcome, as is discussed in a later section, is the Jette Late Life Functional and Disability Instrument (LLFDI) which measures social activity participation in life tasks. The trial workflow is summarized in Fig. 1.

### Inclusion and exclusion criteria

#### Stroke diagnosis

Participants are individuals with recent onset of ischemic or hemorrhagic stroke. For purposes of inclusion in this study, a stroke is defined, according to the World Health Organization definition, as “a rapid onset event of vascular origin reflecting a focal disturbance of cerebral function, excluding isolated impairments of higher function and persisting longer than 24 h [16]”. Stroke diagnosis will be made by a clinician and/or supported by brain imaging.

#### Inclusion criteria

The inclusion criteria for the trial are as follows: (1) age ≥ 40 years, (2) recent stroke (defined as stroke diagnosis occurring within 4 weeks prior to admission into inpatient rehabilitation unit or hospital), (3) able to sit unsupported for 30 s, (4) able to stand on the non-paretic leg for >4 s, (5) able to walk at least 2 m with maximum of 1 person assisting, (6) able to follow a 2-step command, (7) living in the community before stroke and expected to be discharged home, (8) has a caregiver when the patient is doing the exercises for safety reasons, as the tele-therapist is not present when the patient is doing the exercise and a caregiver is needed to catch the patient if he/she should fall.

#### Exclusion criteria

The exclusion criteria for the trial are as follows: (1) has a pacemaker in-situ (because of possible interference of pacemakers by wireless electronic signals), (2) unable to ambulate at least 45 meters (150 feet) prior to stroke, or intermittent claudication while walking less than 200 meters (656 feet), (3) serious cardiac conditions (e.g. hospitalization for myocardial infarction or heart surgery within 3 months, history of severe congestive heart failure, serious and unstable cardiac arrhythmias, hypertrophic cardiomyopathy, severe aortic stenosis, angina or dyspnea at rest or during activities of daily living), (4) history of serious chronic obstructive pulmonary disease or oxygen dependence, (5) severe weight bearing pain, (6) pre-existing neurological disorders such as Parkinson’s disease, amyotrophic lateral sclerosis, multiple sclerosis or severe dementia, (7) history of major head trauma with severe residual deficits, (8) lower extremity amputation, (9) legal blindness or severe visual impairment, (10) severe uncontrolled psychiatric illness such as psychosis, schizophrenia or medication refractory depression (11) life expectancy less than three months, (12) severe arthritis or orthopedic problems that limit passive ranges of motion of lower extremity (e.g. knee flexion contracture of > 10°, knee flexion ROM < 90°, hip flexion contracture > 25°, and ankle plantar flexion contracture > 15°), (13) history of sustained alcoholism or drug abuse in the last six months, (14) hypertension with systolic blood pressure greater than 200 mmHg and diastolic blood pressure greater than 110 mmHg at rest, that cannot be medically controlled into the resting range of 180/100 mmHg.

### Participant recruitment

Participants will be recruited from the inpatient rehabilitation unit of an acute hospital (i.e. SGH) and an inpatient rehabilitation hospital (i.e. AMKTHKH). Each site is expected to recruit approximately 50 participants (25 intervention and 25 controls).

### Screening process

Recruitment will be initiated while the stroke patient is still in the hospital. The research assistant will first screen the medical records for current admissions and medical histories including the list of diseases and conditions at the hospital sites. Once potential patients are identified, the research assistant will approach them and provide information about the study, both in written and verbal form, only after they have indicated their interests. The research assistant has to review the inclusion and exclusion criteria of patients that cannot be determined through medical records.

### Informed consent and enrollment

After eligibility is confirmed, details of study will be explained to potential participant. Once participant agrees to participate, Informed consent is obtained, patient is enrolled and randomization only occurs after enrollment.

### Baseline assessments

The data collected from all participants at baseline are detailed in Table 1. Baseline data collection will be conducted by research assistants who have been trained in administering the questionnaire and assessment tools by the study coordinator. As this study is a single blind (evaluator-blind) randomised controlled trial, research assistants who are involved in the recruitment of participants are not allowed to be involved in the assessment of participants at 3 months and 6 months.

**Table 1:** Baseline assessments

**Personal Information**	**Admission information**	**History of current stroke**
Date of birth	Ward Class	Date of stroke onset
Ethnicity	Date of admission	Type of stroke
Gender	Date of discharge	First or recurrent stroke
Marital Status		Stroke characteristics
		-Side of hemiparesis
		Left
		Right
		-Stroke type
		Infarct
		Bleed
		Both
		Undefined
		-Stroke severity (according to Modified Ranking Scale)
**Co-existing conditions**	**Caregiver**	**Additional tests**
Cardiovascular disease	Spouse	Depression according to Center for Epidemiologic Studies – Depression Scale (CESD-11)
Hypertension	Child	
Peripheral vascular disease	Maid	
Chronic obstructive pulmonary disease	Others	Cognitive function according to Mini-Mental State Exam (MMSE)
Arthritis or other musculoskeletal condition	None	Euro-QOL (EQ-5D)
Diabetes		

#### Depression according to Center for Epidemiologic Studies – Depression Scale (CESD-11)

Informal caregivers will rate each item/symptom on the 11-item Centre for Epidemiologic Studies Depression (CES-D) scale as occurring none/rarely (score = 0), sometimes (score = 1) or often (score = 2) during the past week. Items scores will be summed to obtain a CES-D score (range: 0–18). A previous study from Singapore has reported that the 11-item CES-D has good internal consistency (Cronbach’s alpha = 0.76) and its factor structure matched with that reported in the original validation of the scale. In sensitivity analysis, the CES-D score, a continuous variable, is considered as the outcome.

#### Cognitive function according to Mini-Mental State Exam (MMSE)

The Mini-Mental State Examination (MMSE) is the most commonly used screening tool for cognitive impairment and dementia worldwide. A number of studies have examined the accuracy of the MMSE in the detection of dementia. In Singapore, the localised MMSE version discriminated well between elderly with and without dementia (cut-off 23/24, sensitivity 97.5 %, specificity 75.6 %) [17].

### Standardization

Research assistants and tele-therapists will receive training provided by the study coordinator. The research assistants will be trained in assessing the eligibility of potential participants, obtaining informed consent from participants, randomization process, STARS tele-rehabilitation intervention protocol, the use of tele-rehabilitation system from the patient user interface, patient education on the use of the tele-rehabilitation system (including provision of written instructions to the participant), obtaining outcome measures for both intervention and control groups and documenting the measures. On the other hand, the tele-therapists will be trained in the use of tele-rehabilitation system from the tele-therapist user interface, progression protocols and documenting patients’ progress.

Competency of the tele-therapists and research assistants in their respective responsibilities will be assessed by the study coordinator at the end of the training programme. 90 % competency prior to participating in the study must be attained. To avoid drift, maintenance of competency is important in a multi-site clinical trial. Re-assessment will be done by the study coordinator every 6 months during the study at both sites.

### Randomisation method

Randomization will be conducted after baseline assessment. The participants will be randomized into one of the two groups: tele-rehabilitation intervention and usual care based on equal allocation with randomly varying block sizes of four and six. As the study is conducted in two sites, stratified randomisation will be carried out assuming study site as the stratification factor. The randomisation list is generated using the ***ralloc*** package of Stata 11 (StataCorp LP, College Station, TX, USA).

### Intervention

The tele-rehabilitation intervention has three components: (a) the hardware, (b) the software and (c) the progressive rehabilitative exercises. Prior to the tele-rehabilitation intervention, the research assistant, who previously recruited the participant, will train the participant and their caregiver on the usage of the devices and sensors. The participant and their caregiver will learn how to wear the sensors, operate the hardware and perform all the rehabilitation exercises with the hardware. The training is usually done within 1–3 sessions, with each session being an hour long, until the participant and caregiver are competent in its use. These sessions are done before the discharge of participant and, if necessary, in their homes after discharge. To ensure that the participant/caregiver is able to use the devices and sensors, the participant/caregiver must pass a competency check list, such as switching on the iPad and applying the sensors. Additionally, a baseline visit is scheduled for set-up of tele-rehabilitation system in the participant’s house.

#### Hardware component

The hardware component comprises of an iPad, two limb sensors, a heart rate and blood pressure monitoring set and chargers for these devices. A stable movable floor-mounted flexible iPad stand will be provided to allow easy positioning of iPad to video-record exercises performed by the patient. Engineering details on the tele-rehabilitation system infrastructure and the accuracy of the sensors have been published [18, 19]. Only basic details necessary to understand how the tele-rehabilitation system works are mentioned here.

The limb sensor node is used in the system to determine the orientation of the limb being exercised, which is in turn sent to iPad to determine the number of repetitions and range of motion. Each sensor node consists of an ATMEGA328 microprocessor embedded on a printed circuit Board with three motion sensors and attached to a Bluetooth module. The three motion sensors attached are: ITG3200 (gyroscope) and LSM303DLHC (accelerometer and magnetometer). The Bluetooth module, which is a BLE112-A low energy single mode module produced by Bluegiga tech, is required for wireless communication with iPad.

The data collected from these motion sensors are polled into ATMEGA328 microprocessor through a daisy-chained I^2^C connection. The microprocessor will perform sensor fusion which involves the fusion of data to a 4-dimensional quaternion orientation vector using the algorithm devised by Madgwick [20] to denote the orientation of the sensor with respect to gravity. This is followed by date and time stamping the orientation. The resulting date/time-stamped orientation data is transmitted to the iPad using Bluetooth 4.0 connection. The patient application in the iPad will analyze the data to determine the orientation of the two limb sensors. The orientation data in turn is used to calculate the angle of movement as performed by the participant.

#### Software component

The MySQL relational database management system will be used to store data in the system. The database is used to contain data regarding the patients and therapists such as log-in information. In addition, exercises selected and remotely prescribed by the therapist, and the sensor and video data on the exercises performed by the patients are also stored in the database.

### Patient interface

**Fig. 2:**
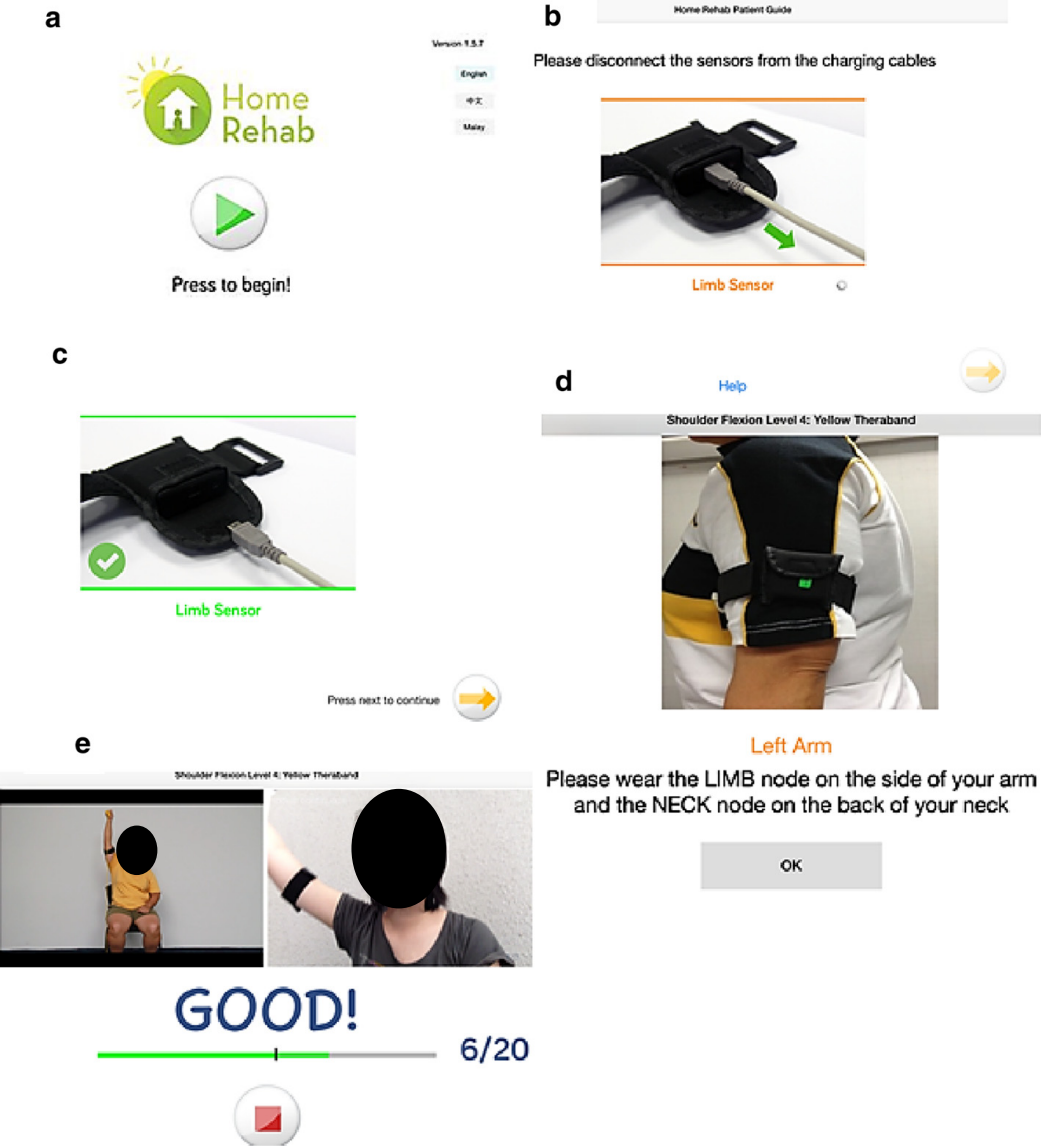
Usage Flow For Patient User Interface. **a** Patient is to press the ‘Play’ button to begin after starting the application. **b** Patient is directed to this page, which prompts him or her to disconnect the limb sensors from the charging cables. After which, the patient is to press the next button in (**c**) to commence. **d** Instruction is provided on how to wear the limb sensors prior to the exercise. **e** Patient is brought to this page to begin their exercise. A demonstration video is provided on the left side of the screen. The patient can look at himself or herself doing the exercises on the right side of the screen. Visual and audio feedback is also provided to patients

The user interface for the patient was designed to be simple and intuitive. A patient guide is provided within the application if the patient needs additional help on its usage. Three languages are provided: English, Chinese and Malay, to cater to the diverse ethnic groups in Singapore. Before the exercises, the iPad needs to be connected to the internet. A log-in is not required as the iPad’s unique identifier is registered to the patient before the initial set-up at the participant’s house (Fig. 2a). After starting the patient application, the application ensures that sensors are detached from the charging cables (Fig. 2b and c) and are properly worn on the patient before commencement of exercises (Fig. 2d). The patient will press the ‘Begin Exercise’ button, followed by the ‘Play button’ in the next screen to commerce the exercises (Fig. 2e). The number of repetitions as performed by the patient is captured and monitored by the sensors. Visual and audio feedback is given to the patient for every repetition that exceeds the target angle set by the therapist.

**Fig. 3:**
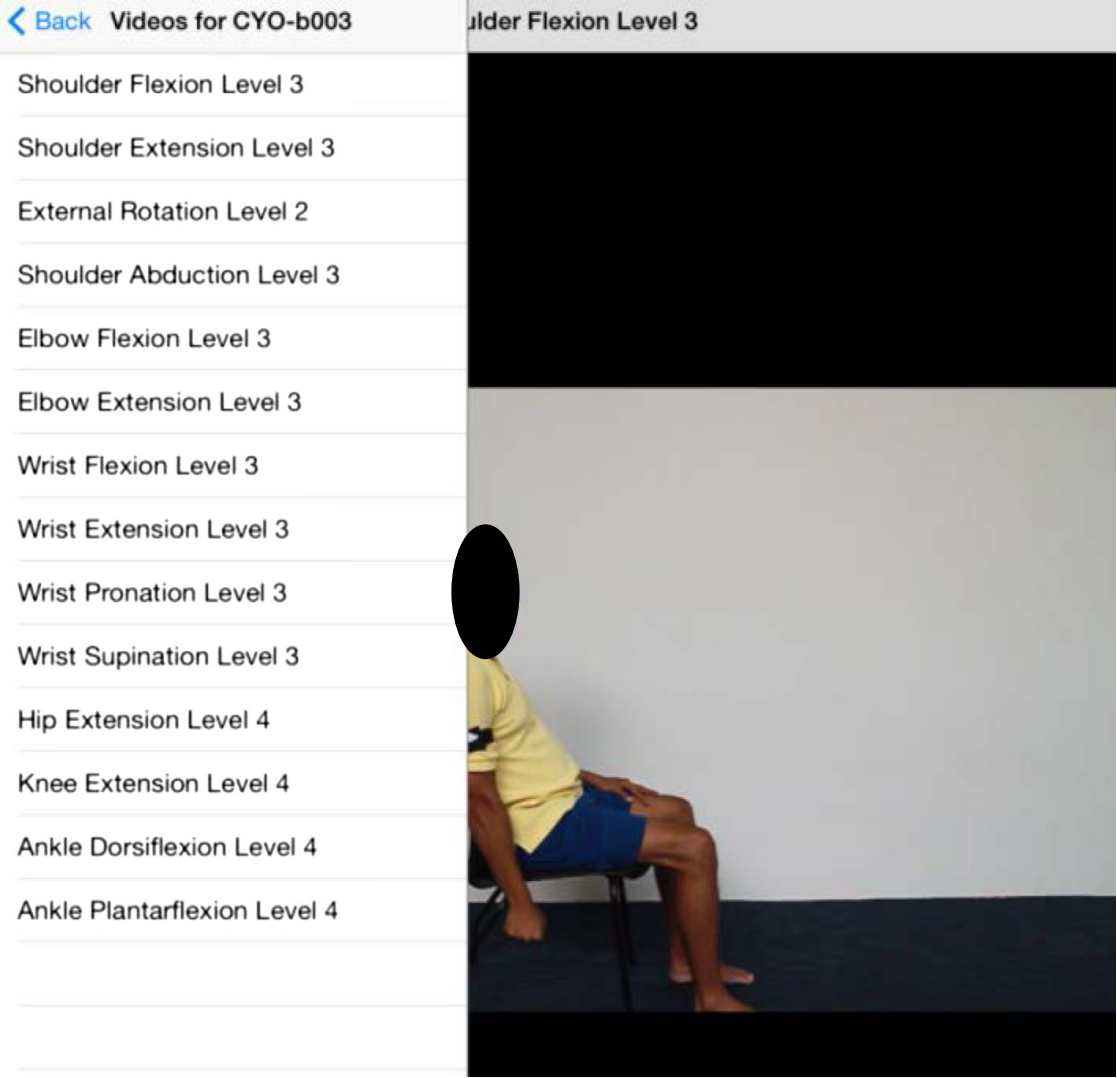
Screenshot of Video Application for Patient. The application allows patients to be familiarized with the exercises before proceeding to the actual exercise regime

Additionally, another application is created for the patients to practise and familiarize themselves with the exercises prescribed prior to the actual exercise regime. Videos of the prescribed exercises are provided in the application (Fig. 3). When the tele-therapists change the prescribed exercises for the patients, the videos in the application will automatically be uploaded onto the patient’s user interface in accordance and sequence to the latest prescription.

### Tele-therapist interface

The user interface for the tele-therapist was designed for efficient display of information on exercises performed by patients. The application allows the tele-therapist to initiate a Facetime video call during a scheduled tele-consultation. On selecting the patient’s name, the tele-therapist will be directed to a page to remotely prescribe exercises and adjust the difficulty (progression) level and the minimum range of motion for each exercise. A summary of the rehabilitative exercises completed by the patient will be presented in graphs for easy interpretation of adherence to prescribed exercise and quality of exercises by the tele-therapist. This is in addition to the videos of patients performing the exercises. The tele-therapist can enter also clinical notes and parameters in the application to record a tele-consultation.

### Web-based data entry

A website was devised for the usage by both the research assistants and the study coordinator. The website allows research assistants to enter baseline data, primary and secondary outcome data, and tele-therapist to document patient’s clinical information during the assessment. Only the study coordinator is allowed to view all patients’ and therapists’ information and update the information in the event of data entry error. Any amendment of data entered will be recorded and date/time-stamped. In addition, the study coordinator can designate therapists and research assistants to the patients. In the occasion when an adverse event happens, the study coordinator will enter the details in the adverse events log in the application.

#### Progressive rehabilitative exercises

The tele-rehabilitation intervention group will receive a standardized rehabilitation programme for three months, which was based on the rehabilitation programme for the control group in a randomized controlled trial on weight-supported locomotion by Duncan et al. [21]. The types of exercises are summarized in Table 2. The programme comprises of both physiotherapy (Category I to IV) and occupational therapy components (Category V). Each tele-session may cover up to all five categories of exercises. The difficulty level and minimum range of motion desired for each exercise prescribed is determined by the tele-therapist who will assess and inform the patient of the change before increasing the difficulty level.

**Table 2:** Progressive rehabilitative exercises for intervention group

Category no.	Category description	Exercise	Levels
I	Upper Limb Strengthening	Shoulder Flexion Shoulder Extension Shoulder External Rotation Shoulder Abduction Elbow Flexion Shoulder Extension Wrist Flexion Wrist Extension Wrist Pronation Wrist Supination Fingers Mass Extension	1. Gravity eliminated, active assisted 2. Gravity eliminated, active 3. Against gravity, no Thera Tube (TT) 4. Against gravity, yellow TT 5. Against gravity, red TT 6. Against gravity, green TT 7. Against gravity, blue TT
II	Lower Limb Strengthening	Hip Flexion Hip Extension Hip Abduction Knee Flexion Knee Extension Ankle Dorsiflexion Ankle Plantarflexion	1. Gravity eliminated, active assisted 2. Gravity eliminated, active 3. Against gravity, no Thera Tube (TT) 4. Against gravity, yellow TT 5. Against gravity, red TT 6. Against gravity, green TT 7. Against gravity, blue TT
III	Seated Balance Exercise		1. Equal weight bearing on ischial tuberosities for 30 seconds 2. Weight shift, lifting alternate leg from chair for 30 seconds 3. Non-paretic Upper Limb: Ipsi-lateral diagonal reaching (anterior and posterior) 4. Paretic Upper Limb: Contra-lateral diagonal reaching (anterior and posterior) 5. Non-paretic Upper Limb: Contra-lateral anterior diagonal reaching (anterior and posterior) 6. Paretic Upper Limb: Ipsi-lateral reaching (anterior and posterior)
IV	Standing Balance Exercise		1. Shoulder- width stance 30 seconds, eyes open, for 30 seconds 2. Feet together 30 seconds, eyes open 3. Staggered stance, Paretic leg in fronton step, eyes open for 30 seconds 4. Staggered stance, Non- paretic leg in fronton step, eyes open for 30 seconds 5. Semi tandem with paretic leg in front for 30 seconds 6. Semi tandem with non-paretic leg in front for 30 seconds
V	Functional Activities	Walking (Lower Limbs)	As much as possible

### Usual care

Stroke patients in Singapore typically receive acute stroke care in an acute hospital, which usually has a dedicated stroke unit. Those who are totally dependent or fully independent in activities of daily living (ADL) usually do not receive post-stroke rehabilitation. Those who are mild to moderately dependent in ADL are usually referred for centre-based rehabilitation after discharge from acute hospital. For those who are moderately to severely dependent in ADL, they usually receive inpatient rehabilitation for 4–6 weeks at inpatient rehabilitation hospitals after discharge from the acute stroke unit. These moderately to severely ADL dependent patients usually would have recovered to become mild to moderately ADL dependent during their inpatient rehabilitation hospitals stay, and hence would be referred for centre-based rehabilitation after being discharged home from inpatient rehabilitation hospitals. In acute hospitals, rehabilitation is usually started soon after stabilization of stroke and would be provided once a day for a total of 1–2 h a day by a physiotherapist and occupational therapist. In inpatient rehabilitation hospitals, rehabilitation is continued upon admission and would be provided twice a day for a total of 2–3 h a day by a physiotherapist and occupational therapist. After discharge, it usually takes 1–2 weeks for centre-based rehabilitation to be initiated and this is usually provided 1–2 times a week for an hour each time.

Home rehabilitation in Singapore is relatively under-developed and generally unavailable and unaffordable for the majority of stroke patients. Government subsidies for acute hospital and inpatient rehabilitation hospital stays are available, and government subsidies for centre-based rehabilitation are also available. However, government subsidies for home rehabilitation, previously unavailable, were only made available mid-way through this trial in July 2014.

It should be noted that for ethical reasons, the intervention group is allowed to supplement their tele-rehabilitation sessions with centre-based rehabilitation if they desire. To control for this at the data analysis stage, we will capture data on the total number of hours of use of centre-based and home rehabilitation for both intervention and control groups throughout the six months from study recruitment.

### Process measures

In this study, exercise done by the participant refers to those that are prescribed by the therapist. Both unsupervised (defined as exercise done by patient without physical presence of a therapist) and supervised exercise (defined as exercise done by patient in the physical presence of a therapist) that participants have performed are tracked during the trial for both intervention and control subjects. Adherence to supervised and unsupervised therapy will be recorded by the subject in a diary to record the number of minutes subject spent each day in therapy with a therapist (excluding time with the tele-therapist) and the number of minutes subject spent each day doing their prescribed rehabilitation exercises. The tele-therapist will check that the intervention subject is entering these data during tele-consultations. For the control (usual care) subjects, the Study Coordinator will call the subject every 4 weeks to check that the intervention subject is entering these data as well.

### Feedback

For intervention participants, feedback about their rehabilitation progress is given via real-time and Facetime. For real-time biofeedback, visual and audio feedback is given to the participant through the iPad for every repetition that exceed the target angle when the participant is performing the rehabilitation exercises. For Facetime, the tele-therapist will have a face-to-face consultation with the participant via video call once a week. They will evaluate the participant based on the data collected from the sensors (which capture the progress of the participant when he/she performs the rehabilitative exercises), and the exercises which the participant may be asked to perform during the video call.

### Outcome assessments

The primary outcome variable and the secondary outcome variables chosen for the study are based on the notion that patients undergoing rehabilitation are more concerned with their improvement in activities and participation instead of reducing their impairment [22]. Although many of the exercises prescribed in this study are known to only reduce impairment, the uses of the primary outcome variable and secondary outcome variables are still valid as the exercises prescribed also improve activity and participation. Furthermore, the participants are asked to walk as much as possible during the 3 months of tele-rehabilitation.

#### Primary outcome variable

Functional ability may be quantified using performance based measures or self-report. Self-report measures remain the most commonly used instruments in many studies involving older adults because of their low cost and practicality [23]. Disability component of the Jette LLFDI, a self-reported measure of social activity participation in life tasks, was selected as the primary outcome measurement for this study, as the same outcome measure was used in a similar tele-rehabilitation study done by Chumbler et al. [13]. The LLFDI will be administered by research assistants. The disability component evaluates self-reported limitations in life activities and frequency of participation in 16 major life tasks categorized under 4 main roles (instrumental, management, social and personal). The degree of limitation and frequency dimensions of each life task are scored on a 0–100 scale, with higher scores indicating higher levels of functioning. The disability component has shown concurrent validity by correlating with the Physical Functioning (PF-10) subscale of the Medical Outcomes Study 36-item Short-Form Health Survey and the London Handicap Scale [24].

#### Secondary outcome variables

There will be 7 secondary outcomes for this trial: (1) gait speed, (2) two-minute walk distance, (3) functional status, (4) balance confidence, (5) patient quality of life, (6) health service utilization and (7) caregiver burden.

### Gait speed

Gait speed is related to the severity of impairment in the home and the community and has been used in many studies [21]. Gait speed will be measured using the timed 5-meter walk test. The individual walks without assistance for 5 meters (16.4 feet) and the time is measured for the intermediate 3 meters (9.8 feet) to allow for acceleration and deceleration. Timing is started when the toes of the leading foot crosses the 1-meter mark and it is stopped when the toes of the leading foot crosses the 4-meter mark. Assistive devices can be used but would be kept consistent and documented from test to test. Three trials are done and the average will be recorded for each session. The participant will be assessed when they are walking at normal comfortable speed and at maximum speed.

### 2-minute walk distance

In addition to the gait speed, the distance a person can walk and the amount of daily walking that a person is able and willing to do are strong indicators of his or her health and condition [21]. Hence, the two-minute walk test will also be conducted. Individuals walk at their usual speed as far as they can without assistance for 2 min and the distance is measured. Assistive devices can be used but would be kept consistent and will be documented from test to test.

### Functional status

Shah-modified BI will be used to assess and compare the improvements in the performance of ADL between the intervention and control groups. The Shah-modified BI is considered to be a valid measurement of ADL, as it is an empirically derived scale with proven inter-observer and test-retest reliability and validity which measures the patient’s functional ability [25]. It involves the assessment of 10-items, which include personal hygiene, bathing self, feeding, toilet, stair climbing, dressing, bowel control, bladder control, ambulation, wheelchair ambulation and chair/bed transfer. Five response options with their score are provided for each item and a total score will be calculated. The score can range from zero to 100 and the higher the score, the more independent the subject is.

### Balance confidence

The 16-items Activities-Specific Balance (ABC) Scale will be used to assess self-perceived efficacy in maintaining balance while performing a number of activities common in community-dwelling older adults such as bending, reaching, and walking both inside and outside the home [26]. This measure has good reliability and internal consistency. Participants have to indicate their level of confidence in doing the activity without losing their balance or becoming unsteady from choosing one of the percentage points on the scale with 0 % representing no confidence and 100 % representing complete confidence. The total rating is calculated and divided by 16 to obtain the overall ABC score. Based on the score, participants can be classified into three categories: < 50 % representing low level of physical functioning, 50-80 % representing moderate level of physical functioning and >80 % representing high level of functioning [26].

### Quality of life

We will use the Euro-QOL (EQ-5D) to measure patients’ quality of life. The EQ-5D is a standardized health-related quality of life questionnaire applicable to a wide range of health conditions and treatments, and it provides a simple descriptive profile and a single index value for health status which can be used in the clinical and economic evaluation of health care [27]. It is designed for self-completion by respondents. It consists of 16-items which measure health state on five dimensions: mobility, self-care, usual activities, pain/discomfort, and anxiety/depression. Each dimension comprises of three levels with their score: no problem = 1, some problem = 2, extreme problem = 3. The result is represented in a 5-digit number, in which each digit shows the level of the respective dimension [27]. Participants will also be asked about their health status and to indicate a value between 0 to 100 where 0 represents the worst health imagined and 100 represented the best health imagined [27]. The EQ-5D will also be administered at baseline for both intervention and control groups in order to calculate change in health-related quality of life before and after the trial which is needed for cost-effectiveness analysis.

### Health service utilization

Singapore Stroke Study Health Service Utilization Form will be used to perform cost-effectiveness analysis in both tele-rehabilitation and usual care. This form is chosen because it has been contextualized to the local Singaporean context. It comprises of 11-items and for each item, utilisation of a particular service is asked. If the answer is “Yes”, the duration and the expenditure of its usage will be asked. Quality-of-life data based on EQ-5D instrument will be combined with resource utilization and cost data to develop incremental cost-effectiveness ratios that will allow investigators to evaluate the cost-effectiveness of tele-rehabilitation compared to usual care [28].

### Caregiver burden

The Zarit Burden Interview (ZBI), which provides a comprehensive assessment of caregiver burden, has been validated in many culturally and ethnically different populations. In this study, the 12-item version of the ZBI will be used with response options ranging from never =1 to nearly always = 5 [29]. The total score will be calculated to determine the burden levels; the higher the score, the greater the burden. The ZBI demonstrates good validity and reliability in measuring the burden of caregivers in Singapore [29] with duration of caregiving and financial problems correlating with greater caregiver burden.

### Research data management and security

All patient information to and from the server will be encrypted via Hypertext Transfer Protocol Secure (HTTPS) which ensures no external parties are able to view it during transfer, a protocol similar to those used by online banking systems. Data access from server is also authenticated by a passphrase which is supplied from the therapist application, ensuring only the patient’s therapist will be able to request and review patient data. Regarding security on the server side, we will be using the Apache server running on a Unix-based operating system and have taken recommended measures to minimise vulnerabilities from hacking.

Only an administrator (in this study, this is the Study Coordinator) will be able to view all patients (both groups) and therapists (intervention group only) data, and update the system in an event of data entry error. The administrator will have privileges to add patients & therapists to the database, pair patients to therapists as well as to update and view all patient data. The independent assessor (blinded evaluator) will be able to input outcome data into the system for both groups at 3 month and 6 month. However, independent assessor will not have access to any participant information other than the latter’s initials and unique study number. The biostatistician will only have read-only access to all the patients’ data for research purposes. However, the biostatistician will not be able to manipulate or distort the data. The biostatistician can also download data for research purposes in the form of an Excel file. All these measures have been put in place to ensure that there is no leakage of patients’ information and only authorized individuals have full access to the secured data.

### Statistical analysis

#### Sample size

Chumbler et al’s paper had similar tele-rehabilitation intervention at 0, 3 and 6 months, and the same primary outcome measure, the Difficulty dimension of the Disability component of the Jette LLFDI [12]. Even though the mean score derived from the results in Chumbler et al’s paper was 11 [12], a mean score of 8 was used in this study due to different geographical and socio-cultural context; Chumbler et al’s study was done in the United States whereas this study was done in Singapore, where rehabilitation is less well-accepted. Hence, based on a two-sided test of 5 % and a power of 90 %, and assuming a difference in mean scores of 8 and a standard deviation of 16, and a correlation of 0.6, a total sample size of around 86 (intervention = 43, controls = 43) was calculated to be adequate. Accounting for 14 % attrition rate, the minimum sample size was determined to be 100 (intervention = 50, controls = 50). It is planned that 100 participants will be recruited within 2 years, after which an additional 6 months is needed for follow-up.

#### Analysis for outcome measures

Data entered will be checked for accuracy at least twice by at least two independent assessors. To understand the structure of missing data and to reduce bias from uncertainty of missing data, multiple imputations will be performed using the Markov Chain Monte Carlo method. All scale-based measures used in this study will be screened via an internal consistency test (Cronbach’s alpha test) before creating a summative score. Furthermore, rigorous validity tests will be performed in two ways: person-centered tests (latent class analysis) and variable-centered tests (exploratory and confirmatory factor analyses).

For descriptive statistics, independent two-sample *t*-test and Analysis of Covariance (ANCOVA) will be performed on all continuous outcome measures (i.e. Shah-modified BI, ABC Scale, gait speed, two-minute walk test and ZBI) between tele-rehabilitation vs. usual care group if outcomes are normally distributed. Mann–Whitney *U* test will be performed on data with skewed distribution to test differences between treatment groups. Chi-square test will be performed on categorical variables. Confounders are age, gender, ethnicity and marital status. Multiple linear regressions will be used to adjust for differences in confounders between intervention and control groups for continuous outcome variables. Ordered logistic regressions will be used to adjust for differences between intervention and control groups for continuous categorical variables. For these multivariate linear and logistic regressions, we will use survey analysis processes with robust standard errors which accounts for nesting effects within patient data as a result of clustering by different recruitment centers. For all statistical analyses, the level of significance (α) will be taken at 0.05. Analyses will be based on the intention-to-treat approach. We will use STATA version 12 for statistical analysis and significance level is set at P < 0.05.

### Cost-effectiveness

Cost effectiveness analyses from both the health system perspective will be performed using the standard quality of life measure EQ-5D and measures of health service utilization and cost over the study period. Quality adjusted life years (QALYs) will be computed for each individual using the EQ-5D score. Incremental cost-effectiveness ratio (ICER) will subsequently be computed to assess incremental costs per QALY gained between treatment and control group. To determine confidence intervals for ICER, a non-parametric bootstrapping method with 1000 replications of the data will be performed. When dominance is observed, full cost analyses will be conducted to determine mean savings per patient.

### Adverse event monitoring and reporting

Adverse events will be carefully monitored during the STARS trial. The teams at the clinical intervention sites will monitor and report all minor or serious adverse events that occur in any participant from the point of enrollment through the 6-month follow-up assessment. Investigators will report to IRB of any adverse events or unanticipated problems, serious or continuing noncompliance, and suspensions or termination.

### Definition of adverse event

An adverse event is defined as any untoward or unfavorable medical occurrence in a human subject, including any abnormal signs, symptoms, or diseases, temporally associated with the subject’s participation in the research, whether or not considered related to the subject’s participation in the research. All adverse events regardless of the seriousness and its relation to the study will be reported to the principal investigator. The research member who observed the adverse event will report the following: the date of onset, action taken with respect to study procedures, corrective treatment/therapy given, outcome and his/her opinion as to whether the adverse event is related to the intervention exercises.

### Definition of unanticipated problem

In general, an unanticipated problem includes any incident, experience, or outcome that meet all the following criteria: (1) unexpected in terms of nature, severity, or frequency given the research procedures that are described in the protocol-related documents and the characteristics of the subject population being studied, (2) related or possibly related to participation in the research and (3) suggestions that the research places subjects or others at a greater risk of harm (including physical, psychological economic, or social harm) than previously known or recognized and requires changes to the research protocol or informed consent document or other corrective actions in order to protect the safety, welfare, or rights of subjects or others. Even though the IRB does not require unanticipated problems to be reported, the principal investigator would be informed of them.

### Reporting of adverse event and unanticipated problem

Adverse events which will be monitored include unplanned re-hospitalization, emergency department visit, death, falls and fractures, recurrent stroke and myocardial infarction. The principal investigator will determine if the adverse event or unanticipated problem has to be reported to IRB such that any corrective changes to the research protocol, informed consent document or other corrective actions are needed. Reporting of any adverse event or unanticipated problem would be done within five working days from the day the principal investigator is aware of the issue.

## Discussion

The STARS Study is a multi-site randomized controlled trial designed to determine the impact of video-conferencing and the use of wearable monitoring devices compared to usual care on the functional recovery of stroke survivors. Stroke results in disabling limitations for patients and supervised rehabilitation can improve functional recovery, but only a minority is undergoing supervised rehabilitation one month after discharge into the community [1]. This trial will make several contributions to the practice of rehabilitation after stroke. Firstly, it will test the practicability of a tele-technology aided rehabilitation intervention on a sufficient number of participants to provide a reasonable basis for confident inference to clinical practice. Secondly, this trial will also test the secondary outcomes of tele-rehabilitation in terms of gait speed, contact time with a therapist, balance, self-reported health-related quality of life, health service utilization and caregiver burden in comparison to usual care. Next, the trial will examine if any greater functional improvement in the intervention group is due to better adherence to supervised exercise or independent of better adherence to supervised exercise. Lastly, the tele-technology aided rehabilitation intervention will be tested against usual care to provide an economic evaluation that incorporates resource utilization and patients’ valuation of their quality of life over the trial period, helping to determine the cost-effectiveness of tele-rehabilitation. Previous studies of the cost of stroke in Singapore estimate only total hospital costs for patients [30] and no published studies has examined the cost-effectiveness of tele-rehabilitation over usual care. Optimal usage of technologies in nationwide post-stroke rehabilitation remains a subject of discussion amongst healthcare decision makers and one of the most paramount problems that stops them from implementing it is due to cost. The trial will provide a start in the process of weighing the clinical and economic costs and benefits.

## Abbreviations

ABC: Activities-specific balance
ADL: Activities of daily living
AMKTHKH: Ang Mo Kio Thye Hua Kwan Hospital
ANCOVA: Analysis of Covariance
BI: Barthel Index
CES-D: Center for Epidemiologic Studies
CESD-11: Centre for Epidemiologic Studies – Depression Scale
CIRB: Centralised Institutional Review Board
DSRB: Domain Specific Review Board
EQ-QOL(Euro-5D): patient self-reported health-related quality-of-life
HTTPS: Hypertext Transfer Protocol Secure
ICER: Incremental cost-effectiveness ratio
IRB: Institutional Review Board
LLFDI: Late Life Functional and Disability Instrument
MMSE: Mini-Mental State Exam
NUS: National University of Singapore
PF-10: Physical functioning
QALYs: Quality adjusted life years
SGH: Singapore General Hospital
STARS: Singapore tele-technology aided rehabilitation in stroke
ZBI: Zarit burden interview
